# Supplementary material for: Biosensor libraries harness large classes of binding domains for construction of allosteric transcriptional regulators
Source: Nat Commun. 2018 Aug 6;9:3101. doi: 10.1038/s41467-018-05525-6 (PMC6079105; doi:10.1038/s41467-018-05525-6)
Supplement: Supplementary file 1 — Supplementary Information [file 41467_2018_5525_MOESM1_ESM.pdf]

SUPPLEMENTARY INFORMATION

Biosensor libraries harness large classes of binding domains for construction of allosteric transcriptional regulators

Juárez *et al.*

Supplementary Note 1

Selection of elements used to construct chimeric TFs

DBD

Table 1 in the main text contains the list of transcriptional repressors whose DBDs were selected for the construction of chimeric TFs. The expanded table Supplementary Table 1 summarizes the key features of the native regulators as well as the exact coordinates of the amino acids chosen as DBD.

The basic criteria for the selection of the individual members from the different families were:

a) Known operator boxes: In parallel to the construction of the chimeric TFs we created a specific reporter plasmid for every DBD. The screening system required a collection of synthetic promoters driving the expression of the reporter gene GFP. The sequence of the operators was vital to use the resulting chimeras as regulatory elements integrated in new genetic circuits.

b) Common domain architecture: We fused all LBDs in the same orientation relative to their natural ligand recognition domain located in the DBDs. To simplify the assembly process, we selected transcriptional repressors with DBD in Nt position.

c) Oligomeric behavior: Most of the chosen DBDs were dimers (or a higher organization of dimers) when bound to DNA. The native proteins undergo a conformational change when the specific inducers for the native repressors bind to their native LBD. The resulting oligomeric change prevents binding to DNA. This phenomenon is a long-standing model of transcriptional repression[1].

d) Availability of a three-dimensional (3D) structure (or that of a close homolog): Crucial to determine the limits of the DBD. Where available, the setting of domain boundaries were guided by existing literature.

All repressors chosen as DBD providers shared a common architecture and their operator boxes had been already identified. However, compromises were made in relation to available 3D structure and oligomeric state in favor of introducing diversity in the DBD collection. In two cases, DeoR and Xre, no empirical evidence of the nature of their oligomeric behavior was available. For Xre its 3D structure is unknown, but the structure of a phylogenetically close relative was available (Supplementary Table 1). A combination of domains representing the gold standard in regulation (e.g. LacI) were selected along with members of lesser known families that presented potentially novel architectures (e.g. Xre).

Several versions of each DBD were integrated in the design of the chimeras. Frequently, there were no studies analyzing the modular nature of the regulators. This lack of information made the 3D structure of the protein the best guide to determine where to impose the limits of the DBD. We implemented a conservative approach based upon avoiding the disruption of stable secondary structures. Different versions of each DBD were included in the library, frequently differing by a number of amino acids in the Ct end of the DBD. In our nomenclature, the smaller version of each DBD was designated as the "CORE" and the longer variants were designated as "ENDS". Supplementary Table 1 contains the coordinates for each variant using the sequences with accession numbers in Table 1 as reference.

LBD

LBDs are key domains that in addition to providing specific sensing capabilities to the TFs, often also contribute to the oligomeric behavior of allosteric TFs[2–4]. The LBDs selected for the proof-of-concept library described in this work were all PBP. Table 2 includes the names and accession numbers of all LBDs used. GGBP (encoded by *mglB*) from *E. coli* was included as an internal positive control, as this is the LBD used in the construction of $SLCP_{GL}$[5] (LacI-GGBP-OD). This domain can form a viable repressor in conjunction with DBD-LacI. However, our objective was to obtain a chimeric TF that was useful as benzoate/4-hydroxybenzoate biosensor. To achieve this objective, the selected LBDs, GGBP aside, all belonged to one of the following 3 groups:

a) PBPs with a ligand partner that had been empirically demonstrated to be benzoate/4-hydroxybenzoate[6,7].
b) PBPs with sequences available in protein databases and with a high percentage of amino acid level identity (PID) with members of the first group (compared by NCBI BLAST). We discarded hits returning a PID between 90 and 100% to introduce more diversity in the library.
c) PBPs closely associated to clusters encoding for benzoate/4-hydroxybenzoate catabolic pathways. ABC transporters presumably specialized in the transport of aromatic compounds are frequently associated to catabolic clusters at a genetic level[8,9].

In their primary sequence, PBP proteins contain signal peptides (SP) for export to the exterior of the inner membrane. We lacked *a priori* information on whether the inclusion of the SP sequence between DBD and LBD would be helpful or detrimental for the transfer of information between domains after binding of the inducer. SP appear to be mostly unstructured except for a short alpha-helix (H region)[10]. While mature PBPs typically lack the SP, $SLCP_{GL}$[5] was constructed using a GGBP gene that preserved most of its sequence (16 out of the 23 codons identified as SP were maintained) and was shown to be functional. A modified version of $SLCP_{GL}$ was assembled as a control chimera, containing the full extent of the SP, and it too was demonstrated to be functional. To increase the diversity of the library all 15 LBD were included in the design as two variants, one containing the SP and another that lacked it, (marked with a "_nSP" in the LBD name). Table 2 contains the coordinates of the region identified as SP according to the tools described in Methods.

LNK

Selection of LBD domains for design of any chimeric protein was relatively straightforward, owing to a set of requirements for functionality of the resulting fusion protein. The selection of linkers (LNK) that give rise to a functional protein, however, is more complicated and often neglected[11]. Despite unpredictable outcomes provided by different linkers when fusing two functional domains, direct

attachment of domains has multiple issues[11] and could have resulted in nonfunctional chimeras. Accordingly, LNKs were critical in the design of the chimeric TFs presented in this work. TF functionality relies upon oligomeric behavior and the successful transfer of information between the LBD and the DBD in the presence/absence of the inducer. This flow of information between the sensing and repressing modules of the protein can be facilitated or completely inhibited by the biophysical properties of the joint linking them. As DBDs are compact domains that in some instances have been proven to themselves bind DNA[12,13], we anticipated that some newly formed chimeras would form super-repressors[14] that were unable to detach from DNA. A TF with a functional DBD and LBD domain might be unresponsive to induction and thus useless if a nonfunctional DBD-LBD joint caused an uncoupling between detection of the inducer and detachment from DNA. To maximize the probability of obtaining functional chimeras, each was constructed in 19 different versions, integrating 18 different LNKs between each DBD-LBD pair, in addition to a version with no LNK. Supplementary Table 2 contains the names, sequence and length of the chosen LNK.

LacI Oligomerization Domain (OD)

There is broad diversity in the way different families of allosteric transcriptional regulators establish contact between the monomers to acquire their quaternary structure. In some cases the main contact surfaces are located in the DBD[15–17], whereas in other families diffused or localized contacts in the LBD are crucial[18–20]. The intensive study of LacI has allowed the identification at Ct of LBD of a so-called oligomerization domain (OD) responsible for the establishment of surface contacts when LacI oligomerizes. Selected amino acids in OD are responsible for the association of two LacI dimers, consolidating a tetrameric quaternary structure which would be more stable than the dimer[21,22]. Since PBPs are closely related to LBDs of regulator families such as LacI/GalR, we hypothesized they could potentially provide some of the surface contacts required for chimera oligomerization. However, the PBPs included in the chimeras have not undergone an evolutionary process selecting for improved cooperativity between monomers in the LBD interface. To increase the probability of chimeric monomers forming a

stable quaternary structure, we created a variant of each chimeric TF that would be translationally fused to LacI OD. The potential of this approach has been suggested previously during construction of a glucose-responsive chimera $SLCP_{GL}$[5], however, no comparison of protein functionality with and without OD has been performed.

Supplementary Methods

eLCR: types of chimeras based on the number and nature of oligonucleotides necessary for their assembly

The eLCR technique presented in this paper was based on the use of small ssDNA fragments, as it was the case for canonical LCR[23]. However, in eLCR the oligonucleotides were tasked not only with the stapling effect in scarless ligation of two DNA fragments but also with the introduction of extra sequences in between the DBD and LBD domains.

Oligonucleotides with the same sequence and orientation as the template strand were designated as Infra whereas those corresponding to the coding strand were designated as Supra. Infra and Supra oligonucleotides served two different purposes in the assembly process. Infra oligonucleotides acted as staples connecting DBD and LBD but in addition they were also a scaffold for the incorporation of new sequences between the two domain they connected. The Infra oligonucleotides incorporated reverse-complementary sequences to the new codons to be introduced between DBDs and LBDs. The Supra oligonucleotides contained those extra codons, so that in the assembly process they were docked in place using the Infra as scaffold.

Four distinct kinds of chimeras were defined depending on the number of bases needed between DBD-Ct and Nt-LBD. The length of the sequence introduced between DBD and LBD defined the amount and nature of Infra and Supra oligonucleotides required for the assembly of any given chimera (Fig. 2b):

a) **Class I (0 b; 1 Infra):** Chimeras formed by the ligation of the DBD-CORE sequences and the LBD (both with and without signal peptides). These constructions benefited from a standard LCR when performing the assembly. They represented 0.64% of the total library. The Infra staple oligonucleotide contains 30 b of homology with each DNA fragment.

b) **Class II (3-18; 1 long Infra, 1 Supra)**: The chimeras in this group were assembled by the longest Infra staples of the library (ranging between 115 and 130 b). These Infra staples contained a 56 b extended homology region to the DBD and another 56 b to the LBD. These elongated Infra oligonucleotides allowed the introduction of 3 to 18 b between the DBD and LBD. The efficiency of incorporating a 3-18 b long Supra oligonucleotide would be extremely low. Using an Infra with longer homology to DBD and LBD enabled the 3 to 18 b linking the domains to be flanked by sequences identical to the 3´-end of DBD and the 5´-end of LBD. As a result, these Supra oligonucleotides had a Tm that facilitated their hybridization to the Infra within a similar range than the rest of the Supra. The CORE-ENDS and/or LNKs would have been too short to efficiently hybridize by themselves. Class II chimeras represent the 22.29% of the library.

c) **Class III (21-66; 1 Infra, 1 Supra):** Chimeras assembled through an Infra oligonucleotide with 30 b of homology to each one of the two domains it connects. A Supra oligonucleotide introduced the DBD-ENDS and/or LNK. These represented 44.30% of the library.

d) **Class IV (69-243; 3 Infra, 2 Supra):** Here, the sequences that needed to be introduced between DBD and LBD were so long that it we split them to fall within the range of DNA lengths that can be confidently synthesized in microarray manufacturing (Methods). Two Supra oligonucleotides were required, as well as three Infra: one of them stapling LBD-Supra1, another one stapling Supra1-Supra2 and a final one to Supra2-LBD. These fusion genes represented the 32.77% of the library.

Construction of expression vectors (pCKTRBS/pCKTRBS-OD)

Plasmid name: pCKTRBS/pCKTRBS-OD

Addgene IDs: #102923 / #102924

Abbreviations:

- *p*: Plasmid.
- *CK*: pCK01 derivative.
- *T*: Anhydrotetracycline (aTc) responsive promoter (*PtetO*).
- *RBS*: Contains a consensus RBS downstream of the *PtetO* promoter.
- *OD*: pCKTRBS-OD contains the Oligomerization Domain (OD) of LacI.

Plasmid chassis: pCK01 (Supplementary Data 1).

Plasmid components (Coordinates for pCKTRBS-OD):

- pCK01 [1-2017]:

  oripSC101 [216-470]

- pKD154 [2018-2704]:

  tetR (reverse strand) [2018-2644]

  PtetR (reverse strand) [2655-2694]. ***-10***, ***-35*** boxes. Modified in order to disrupt overlapping operator boxes for the promoter driving *tetA* expression in the original vector:

  Original *PtetR* (5´>3´):

  TGGTAAAATAACTCTATCAATGA***TAGAGT***GTCAACAAAAATTAGGAATTAATG

  Modified *PtetR* (5´>3´):

  TGGTAAAGGCACGCTATCAATGA***TAGAGT***AGACCCAAAAATT***AGGAA***TTAATG

  Spacer [2695-2704] from a sequence upstream of *tetR* on pKD154

- *PtetO* [2705-2784] Modified ***-10***, ***-35*** boxes. ***TetR boxes***. The sequence for *PtetO* was obtained from pZE21-MCS and ordered as a synthetic fragment (see below).
- Synthetic consensus **RBS** with STOP codons in 3 frames [2817-2835]

- Oligomerization Domain of LacI (LacI OD) [2836-2946]
- pCK01 [2947-4158]:

  Chloramphenicol acetyl-transferase, Cm^R (reverse strand) [3287-3946]

Construction details: Oligonucleotide sequences shown in Supplementary Data 2.

a) The chassis for the construction of pCKTRBS and pCKTRBS-OD is the intermediate plasmid pCK01-MlyI_free, a pCK01 vector in which restriction sites for MlyI were removed. pCK01-MlyI_free was constructed by Gibson assembly of two DNA fragments that when stuck together reconstituted a pCK01 lacking the restriction sites. These fragments were amplified with the primer pairs pCK01-MlyI_free_North_F3 / pCK01-MlyI_free_North_R3 and pCK01-MlyI_free_South_F3 / pCK01-MlyI _free_South_R3.

b) Using pKD154 as a template, a *teR-PtetR* fragment was amplified using the primer pair pKD154_upstream-tetR_F1 / pKD154_tetR-pCK01_R1.

c) In parallel, a synthetic DNA fragment (*3tetR-PtetR-PtetO-pCK*) containing the *PtetO* sequence flanked by homology arms to the *tetR-PtetR* fragment and to the pCK01-MlyI_free chassis was obtained from IDT (Coralville, IA):

CTTTACTTTTATCTAATCTAGACATCATTAATTCCTAATTTTTGGGTCT***ACTCTA***TCATTGATAGCGTGCCT***TTACCA***CTC

CCTATCAtcgttt***ccctatcagtgatagaga******ttgaca***t***ccctatcagtgatagaga******tact***gagcacatcagcaggacgcac

tgaccgGCGGCCGCGCGAATTCGAGC

The synthetic DNA fragment *3tetR-PtetR-PtetO-pCK* was amplified with the primer pair 3tetR_R1 / pCK01_R2.

d) The intermediate vector pCKT was the result of a three-way Gibson assembly of the amplified *tetR-PtetR* and *3tetR-PtetR-PtetO-pCK* cassettes together with a linearized pCK01-MlyI_free amplified with the divergent primers pCK01_F1/pCK01_R1, that excluded the the *Plac* promoter from the vector amplification.

e) pCKTRBS was constructed by divergent amplification of pCKT using the primers pCKPolyF1_rv_RBS-Poly / pCKT_RBS-Poly_5’. These primers introduced tails that reconstituted a consensus RBS downstream of *PtetO* when the plasmid was re-circularized by ligation.

d) pCKTRBS-OD was constructed following the same principle used for pCKTRBS. A PCR product of pCKTRBS amplified with the primer pair pCKT_RBS-OD_3’/pCKT_Poly-OD_5’ contained tails that reconstituted a LacI OD to generate pCKTRBS-OD when the plasmid was re-circularized by ligation.

> pCKTRBS-OD

CTGTAGTGCCATTTACCCCCATTCACTGCCAGAGCCGTGAGCGCAGCGAACTGAATGTCACGAAAAAGACAGCGACTTAGGTGCCT
GATGGTCGGAGACAAAAGGAATATTCAGCGATTTGCCCGAGCTTGCGAGGGTGCTACTTAAGCCTTTAGGGTTTTAAGGTCTGTTT
TGTAGAGGAGCAAACAGCGTTTGCGACATCCTTTTGTAATACTGCGGAACTGACTAAAGTAGTGAGTTATACACAGGGCTGGGATC
TATTCTTTTTATCTTTTTTTATTCTTTCTTTATTCTATAAATTATAACCACTTGAATATAAACAAAAAAAACACACAAAGGTCTAG
CGGAATTTACAGAGGGTCTAGCAGAATTTACAAGTTTTCCAGCAAAGGTCTAGCAGAATTTACAGATACCCACAACTCAAAGGAAA
AGGACTAGTAATTATCATTGACTAGCCCATCTCAATTGGTATAGTGATTAAAATCACCTAGACCAATTGAGATGTATGTCTGAATT
AGTTGTTTTCAAAGCAAATGAACTAGCGATTAGTCGCTATGACTTAACGGAGCATGAAACCAAGCTAATTTTATGCTGTGTGGCAC
TACTCAACCCCACGATTGAAAACCCTACAAGGAAAGAACGGACGGTATCGTTCACTTATAACCAATACGCTCAGATGATGAACATC
AGTAGGGAAAATGCTTATGGTGTATTAGCTAAAGCAACCAGAGAGCTGATGACGAGAACTGTGGAAATCAGGAATCCTTTGGTTAA
AGGCTTTGAGATTTTCCAGTGGACAAACTATGCCAAGTTCTCAAGCGAAAAATTAGAATTAGTTTTTAGTGAAGAGATATTGCCTT
ATCTTTTCCAGTTAAAAAAATTCATAAAATATAATCTGGAACATGTTAAGTCTTTTGAAAACAAATACTCTATGAGGATTTATGAG
TGGTTATTAAAAGAACTAACACAAAAGAAAACTCACAAGGCAAATATAGAGATTAGCCTTGATGAATTTAAGTTCATGTTAATGCT
TGAAAATAACTACCATGAGTTTAAAAGGCTTAACCAATGGGTTTTGAAACCAATAAGTAAAGATTTAAACACTTACAGCAATATGA
AATTGGTGGTTGATAAGCGAGGCCGCCCGACTGATACGTTGATTTTCCAAGTTGAACTAGATAGACAAATGGATCTCGTAACCGAA
CTTGAGAACAACCAGATAAAAATGAATGGTGACAAAATACCAACAACCATTACATCAGATTCCTACCTACATAACGGACTAAGAAA
AACACTACACGATGCTTTAACTGCAAAAATTCAGCTCACCAGTTTTGAGGCAAAATTTTTGAGTGACATGCAAAGTAAGTATGATC
TCAATGGTTCGTTCTCATGGCTCACGCAAAAACAACGAACCACACTAGAGAACATACTGGCTAAATACGGAAGGATCTGAGGTTCT
TATGGCTCTTGTATCTATCAGTGAAGCATCAAGACTAACAAACAAAAGTAGAACAACTGTTCACCGTTACATATCAAAGGGAAAAC
TGTCCATATGCACAGATGAAAACGGTGTAAAAAAGATAGATACATCAGAGCTTTTACGAGTTTTTGGTGCATTTAAAGCTGTTCAC
CATGAACAGATCGACAATGTAACAGATGAACAGCATGTAACACCTAATAGAACAGGTGAAACCAGTAAAACAAAGCAACTAGAACA
TGAAATTGAACACCTGAGACAACTTGTTACAGCTCAACAGTCACACATAGACAGCCTGAAACAGGCGATGCTGCTTATCGAATCAA
AGCTGCCGACAACACGGGAGCCAGTGACGCCTCCCGTGGGGAAAAAATCATGGCAATTCTGGAAGAAATAGCGCCCAATACGCAAA
CCGCCTCTCCCCGCGCGTTGGCCGATTCATTAATGCAGCTGGCACGACAGGTTTCCCGACTGGAAAGCGGGCAGTGAGCGCAACGC
AATTAATGTGAGTTAGCTCACTCATTAGGCACCCCAGGC[redacted]TAATTCCTAATTTTTGGGTCTA
CTCTATCATTGATAGCGTGCCTTTACCACTCCCTATCATCGTTTCCCTATCAGTGATAGAGATTGACATCCCTATCAGTGATAGAG
ATACTGAGCACATCAGCAGGACGCACTGACCGGCGGCCGCGCGAATTCGAGCTCGGTACCCGGGTGACCTAAGGAGGTAAATAAAA
AGAAAAACCACCCTGGCGCCCAATACGCAAACCGCCTCTCCCCGCGCGTTGGCCGATTCATTAATGCAGCTGGCACGACAGGTTTC
CCGACTGGAAAGCGGGCAGTGAGATCCTCTAGAGTGGACCTGCAGGCATGCAAGCTTGCGGCCGCCCGTTTTTATCAGGCTCTGGG
AGGCAGAATAAATGATCATATCGTCAATTATTACCTCCACGGGGAGAGCCTGAGCAAACTGGCCTCAGGCATTTGAGAAGCACACG

GTCACACTGCTTCCGGTAGTCAATAAACCGGTAAACCAGCAATAGACATAAGCGGCTATTTAACGACCCTGCCCTGAACCGACGAC
CGGGTCGAATTTGCTTTCGAATTTCTGCCATTCATCCGCTTATTATCACTTATTCAGGCGTAGCAACCAGGCGTTTAAGGGCACCA
ATAACTGCCTTAAAAAAATTACGCCCCGCCCTGCCACTCATCGCAGTACTGTTGTAATTCATTAAGCATTCTGCCGACATGGAAGC
CATCACAAACGGCATGATGAACCTGAATCGCCAGCGGCATCAGCACCTTGTCGCCTTGCGTATAATATTTGCCCATGGTGAAAACG
GGGGCGAAGAAGTTGTCCATATTGGCCACGTTTAAATCAAAACTGGTGAAACTCACCCAGGGATTGGCTGAGACGAAAAACATATT
CTCAATAAACCCTTTAGGGAAATAGGCCAGGTTTTCACCGTAACACGCCACATCTTGCGAATATATGTGTAGAAACTGCCGGAAAT
CGTCGTGGTATTCACTCCAGAGCGATGAAAACGTTTCAGTTTGCTCATGGAAAACGGTGTAACAAGGGTGAACACTATCCCATATC
ACCAGCTCACCGTCTTTCATTGCCATACGAAATTCCGGATGAGCATTCATCAGGCGGGCAAGAATGTGAATAAAGGCCGGATAAAA
CTTGTGCTTATTTTTCTTTACGGTCTTTAAAAAGGCCGTAATATCCAGCTGAACGGTCTGGTTATAGGTACATTGAGCAACTGACT
GAAATGCCTCAAAATGTTCTTTACGATGCCATTGGGATATATCAACGGTGGTATATCCAGTGATTTTTTTCTCCATTTTAGCTTCC
TTAGCTCCTGAAAATCTCGATAACTCAAAAAATACGCCCGGTAGTGATCTTATTTCATTATGGTGAAAGTTGGAACCTCTTACGTG
CCGATCAACGTCTCATTTTCGCCAAAAGTTGGCCCAGGGCTTCCCGGTATCAACAGGGACACCAGGATTTATTTATTCTGCGAAGT
GATCTTCCGTCACAGGTATTTATTCGGCGC

> pCKTRBS

CTGTAGTGCCATTTACCCCCATTCACTGCCAGAGCCGTGAGCGCAGCGAACTGAATGTCACGAAAAAGACAGCGACTTAGGTGCCT
GATGGTCGGAGACAAAAGGAATATTCAGCGATTTGCCCGAGCTTGCGAGGGTGCTACTTAAGCCTTTAGGGTTTTAAGGTCTGTTT
TGTAGAGGAGCAAACAGCGTTTGCGACATCCTTTTGTAATACTGCGGAACTGACTAAAGTAGTGAGTTATACACAGGGCTGGGATC
TATTCTTTTTATCTTTTTTTATTCTTTCTTTATTCTATAAATTATAACCACTTGAATATAAACAAAAAAAACACACAAAGGTCTAG
CGGAATTTACAGAGGGTCTAGCAGAATTTACAAGTTTTCCAGCAAAGGTCTAGCAGAATTTACAGATACCCACAACTCAAAGGAAA
AGGACTAGTAATTATCATTGACTAGCCCATCTCAATTGGTATAGTGATTAAAATCACCTAGACCAATTGAGATGTATGTCTGAATT
AGTTGTTTTCAAAGCAAATGAACTAGCGATTAGTCGCTATGACTTAACGGAGCATGAAACCAAGCTAATTTTATGCTGTGTGGCAC
TACTCAACCCCACGATTGAAAACCCTACAAGGAAAGAACGGACGGTATCGTTCACTTATAACCAATACGCTCAGATGATGAACATC
AGTAGGGAAAATGCTTATGGTGTATTAGCTAAAGCAACCAGAGAGCTGATGACGAGAACTGTGGAAATCAGGAATCCTTTGGTTAA
AGGCTTTGAGATTTTCCAGTGGACAAACTATGCCAAGTTCTCAAGCGAAAAATTAGAATTAGTTTTTAGTGAAGAGATATTGCCTT
ATCTTTTCCAGTTAAAAAAATTCATAAAATATAATCTGGAACATGTTAAGTCTTTTGAAAACAAATACTCTATGAGGATTTATGAG
TGGTTATTAAAAGAACTAACACAAAAGAAAACTCACAAGGCAAATATAGAGATTAGCCTTGATGAATTTAAGTTCATGTTAATGCT
TGAAAATAACTACCATGAGTTTAAAAGGCTTAACCAATGGGTTTTGAAACCAATAAGTAAAGATTTAAACACTTACAGCAATATGA
AATTGGTGGTTGATAAGCGAGGCCGCCCGACTGATACGTTGATTTTCCAAGTTGAACTAGATAGACAAATGGATCTCGTAACCGAA
CTTGAGAACAACCAGATAAAAATGAATGGTGACAAAATACCAACAACCATTACATCAGATTCCTACCTACATAACGGACTAAGAAA
AACACTACACGATGCTTTAACTGCAAAAATTCAGCTCACCAGTTTTGAGGCAAAATTTTTGAGTGACATGCAAAGTAAGTATGATC
TCAATGGTTCGTTCTCATGGCTCACGCAAAAACAACGAACCACACTAGAGAACATACTGGCTAAATACGGAAGGATCTGAGGTTCT
TATGGCTCTTGTATCTATCAGTGAAGCATCAAGACTAACAAACAAAAGTAGAACAACTGTTCACCGTTACATATCAAAGGGAAAAC
TGTCCATATGCACAGATGAAAACGGTGTAAAAAAGATAGATACATCAGAGCTTTTACGAGTTTTTGGTGCATTTAAAGCTGTTCAC
CATGAACAGATCGACAATGTAACAGATGAACAGCATGTAACACCTAATAGAACAGGTGAAACCAGTAAAACAAAGCAACTAGAACA
TGAAATTGAACACCTGAGACAACTTGTTACAGCTCAACAGTCACACATAGACAGCCTGAAACAGGCGATGCTGCTTATCGAATCAA
AGCTGCCGACAACACGGGAGCCAGTGACGCCTCCCGTGGGGAAAAAATCATGGCAATTCTGGAAGAAATAGCGCCCAATACGCAAA
CCGCCTCTCCCCGCGCGTTGGCCGATTCATTAATGCAGCTGGCACGACAGGTTTCCCGACTGGAAAGCGGGCAGTGAGCGCAACGC
AATTAATGTGAGTTAGCTCACTCATTAGGCACCCCAGGCTTAAGACCCACTTTCACATTTAAGTTGTTTTTCTAATCCGCATATGA
TCAATTCAAGGCCGAATAAGAAGGCTGGCTCTGCACCTTGGTGATCAAATAATTCGATAGCTTGTCGTAATAATGGCGGCATACTA
TCAGTAGTAGGTGTTTCCCTTTCTTCTTTAGCGACTTGATGCTCTTGATCTTCCAATACGCAACCTAAAGTAAAATGCCCCACAGC
GCTGAGTGCATATAATGCATTCTCTAGTGAAAAACCTTGTTGGCATAAAAAGGCTAATTGATTTTCGAGAGTTTCATACTGTTTTT
CTGTAGGCCGTGTACCTAAATGTACTTTTGCTCCATCGCGATGACTTAGTAAAGCACATCTAAAACTTTTAGCGTTATTACGTAAA
AAATCTTGCCAGCTTTCCCCTTCTAAAGGGCAAAAGTGAGTATGGTGCCTATCTAACATCTCAATGGCTAAGGCGTCGAGCAAAGC
CCGCTTATTTTTTACATGCCAATACAATGTAGGCTGCTCTACACCTAGCTTCTGGGCGAGTTTACGGGTTGTTAAACCTTCGATTC
CGACCTCATTAAGCAGCTCTAATGCGCTGTTAATCACTTTACTTTTATCTAATCTAGACATCAT*TAATTCCTAATTTTTGGGTCT**A**
CTCTATCATTGATAGCGTGCCT**TTACCA***CTCCCTATCATCGTTT***CCCTATCAGTGATAGAGATTGACA**T**CCCTATCAGTGATAGAG
ATACT**GAGCACATCAGCAGGACGCACTGACCGGCGGCCGCGCGAATTCGAGCTCGGTACCCGGGTGACCTAAGGAGGTAAATAGAT
CCTCTAGAGTGGACCTGCAGGCATGCAAGCTTGCGGCCGCCCGTTTTTATCAGGCTCTGGGAGGCAGAATAAATGATCATATCGTC
AATTATTACCTCCACGGGGAGAGCCTGAGCAAACTGGCCTCAGGCATTTGAGAAGCACACGGTCACACTGCTTCCGGTAGTCAATA
AACCGGTAAACCAGCAATAGACATAAGCGGCTATTTAACGACCCTGCCCTGAACCGACGACCGGGTCGAATTTGCTTTCGAATTTC
TGCCATTCATCCGCTTATTATCACTTATTCAGGCGTAGCAACCAGGCGTTTAAGGGCACCAATAACTGCCTTAAAAAAATTACGCC
CCGCCCTGCCACTCATCGCAGTACTGTTGTAATTCATTAAGCATTCTGCCGACATGGAAGCCATCACAAACGGCATGATGAACCTG
AATCGCCAGCGGCATCAGCACCTTGTCGCCTTGCGTATAATATTTGCCCATGGTGAAAACGGGGGCGAAGAAGTTGTCCATATTGG

CCACGTTTAAATCAAAACTGGTGAAACTCACCCAGGGATTGGCTGAGACGAAAAACATATTCTCAATAAACCCTTTAGGGAAATAG
GCCAGGTTTTCACCGTAACACGCCACATCTTGCGAATATATGTGTAGAAACTGCCGGAAATCGTCGTGGTATTCACTCCAGAGCGA
TGAAAACGTTTCAGTTTGCTCATGGAAAACGGTGTAACAAGGGTGAACACTATCCCATATCACCAGCTCACCGTCTTTCATTGCCA
TACGAAATTCCGGATGAGCATTCATCAGGCGGGCAAGAATGTGAATAAAGGCCGGATAAAACTTGTGCTTATTTTTCTTTACGGTC
TTTAAAAAGGCCGTAATATCCAGCTGAACGGTCTGGTTATAGGTACATTGAGCAACTGACTGAAATGCCTCAAAATGTTCTTTACG
ATGCCATTGGGATATATCAACGGTGGTATATCCAGTGATTTTTTTCTCCATTTTAGCTTCCTTAGCTCCTGAAAATCTCGATAACT
CAAAAAATACGCCCGGTAGTGATCTTATTTCATTATGGTGAAAGTTGGAACCTCTTACGTGCCGATCAACGTCTCATTTTCGCCAA
AAGTTGGCCCAGGGCTTCCCGGTATCAACAGGGACACCAGGATTTATTTATTCTGCGAAGTGATCTTCCGTCACAGGTATTTATTC
GGCGC

Construction pHC_DYOLacI-R and derivative plasmids (pHC_DYO*DBD*-R)

Plasmid name: pHC_DYOLacI-R

Addgene ID: #102922

Abbreviations:

- *p*: Plasmid.
- *HC*: High-Copy number.
- *DYO*: Design Your Own.
- *LacI*: The plasmid is a reporter for chimeric TF carrying DBD-LacI.
- *R*: Contains a protein fluorescent in the Red (mCherry) that may serve as a reporter of the per cell copy-number of the plasmid to normalize certain experiments.

Plasmid chassis: pUC19 (Supplementary Data 1).

Plasmid components:

- pUC19 [1-2279]:
  - *Pbla*, ***bla*** (ampicillin/carbenicillin resistance) [215-1206]
  - pMB1 derived replication origin [1371-1965]
  - Homology arm for Gibson assembly [2260-2279]
- pGERC [2280-2990]:
  - mCherry (reverse strand) [2280-2990]

- pIZ1016 [2991-3500]

  Ptac [3446-3500] ***-35***, ***-10***, ***+1***, *lac operator*. This region shadowed in yellow was substituted by the different synthetic promoters listed in Supplementary Data 3 for the construction of the pHC_DYO*DBD*-R family of vectors.

- Synthetic consensus **RBS** with STOP codons in 3 frames [3515-3533]
- pGERC [3534-4250]:

  sfGFP [3534-4250]

Construction details: Oligonucleotide sequences shown in Supplementary Data 2. The gene encoding mCherry in pGERC was amplified using the primer pair mCherryRFP_Sri pIZ 5' / mCherryRFP_Sri pIZ 3' whereas the gene encoding sfGFP was amplified using sfGFP_Sri pIZPtac-RBS 5' / sfGFP_Sri pIZ 3'. Two regions of the intermediate cloning vector pIZ1016 were amplified using the primer pairs pIZ1016 F3 / pIZ1016 R3 and pIZ1016 F2 / pIZ1016 R4. The mCherry and sfGFP fragments contained 20-bp flanking homology arms so that the 4 fragments could be Gibson assembled in the following way: F2/R4-mCherry-F3/R3- sfGFP-F2/R4. The resulting plasmid was named pDYOLacI-R. In order to obtain the high-copy number version of this construction, pHC_DYOLacI-R, the reporter regions compressed between the genes encoding mCherry and sfGFP was PCR amplified with the primer pair lacIQ-pUC-Gibson 5' / sfGFP-pUC-Gibson 3', that included 20 b flanking homology arms to the pUC19 chassis (antibiotic resistance and replication origin) amplified using the primer pair pUC F1 / pUC R1. Both PCR fragments were Gibson assembled and transformed into *E. coli* NEB5-alpha *F´ I*^{q}.

> pHC_DYOLacI-R

TTAAGCCAGCCCCGACACCCGCCAACACCCGCTGACGCGCCCTGACGGGCTTGTCTGCTCCCGGCATCCGCTTACAGACAAGCTGT
GACCGTCTCCGGGAGCTGCATGTGTCAGAGGTTTTCACCGTCATCACCGAAACGCGCGAGACGAAAGGGCCTCGTGATACGCCTAT
TTTTATAGGTTAATGTCATGATAATAATGGTTTCTTAGACGT*CAGGTGGCACTTTTCGGGGAAATGTGCGCGGAACCCCTATTTGT*
TTATTTTTCTAAATACATTCAAATATGTATCCGCTCATGAGACAATAACCCTGATAAATGCTTCAATAATATTG ***AAAAAGGAAGAG***
TATGAGTATTCAACATTTCCGTGTCGCCCTTATTCCCTTTTTTGCGGCATTTTGCCTTCCTGTTTTTGCTCACCCAGAAACGCTGG
TGAAAGTAAAAGATGCTGAAGATCAGTTGGGTGCACGAGTGGGTTACATCGAACTGGATCTCAACAGCGGTAAGATCCTTGAGAGT
TTTCGCCCCGAAGAACGTTTTCCAATGATGAGCACTTTTAAAGTTCTGCTATGTGGCGCGGTATTATCCCGTATTGACGCCGGGCA
AGAGCAACTCGGTCGCCGCATACACTATTCTCAGAATGACTTGGTTGAGTACTCACCAGTCACAGAAAAGCATCTTACGGATGGCA
TGACAGTAAGAGAATTATGCAGTGCTGCCATAACCATGAGTGATAACACTGCGGCCAACTTACTTCTGACAACGATCGGAGGACCG
AAGGAGCTAACCGCTTTTTTGCACAACATGGGGGATCATGTAACTCGCCTTGATCGTTGGGAACCGGAGCTGAATGAAGCCATACC
AAACGACGAGCGTGACACCACGATGCCTGTAGCAATGGCAACAACGTTGCGCAAACTATTAACTGGCGAACTACTTACTCTAGCTT

CCCGGCAACAATTAATAGACTGGATGGAGGCGGATAAAGTTGCAGGACCACTTCTGCGCTCGGCCCTTCCGGCTGGCTGGTTTATT
GCTGATAAATCTGGAGCCGGTGAGCGTGGGTCTCGCGGTATCATTGCAGCACTGGGGCCAGATGGTAAGCCCTCCCGTATCGTAGT
TATCTACACGACGGGGAGTCAGGCAACTATGGATGAACGAAATAGACAGATCGCTGAGATAGGTGCCTCACTGATTAAGCATTGGT
AACTGTCAGACCAAGTTTACTCATATATACTTTAGATTGATTTAAAACTTCATTTTTAATTTAAAAGGATCTAGGTGAAGATCCTT
TTTGATAATCTCATGACCAAAATCCCTTAACGTGAGTTTTCGTTCCACTGAGCGTCAGACCCCGTAGAAAAGATCAAAGGATCTTC
TTGAGATCCTTTTTTTCTGCGCGTAATCTGCTGCTTGCAAACAAAAAAACCACCGCTACCAGCGGTGGTTTGTTTGCCGGATCAAG
AGCTACCAACTCTTTTTCCGAAGGTAACTGGCTTCAGCAGAGCGCAGATACCAAATACTGTTCTTCTAGTGTAGCCGTAGTTAGGC
CACCACTTCAAGAACTCTGTAGCACCGCCTACATACCTCGCTCTGCTAATCCTGTTACCAGTGGCTGCTGCCAGTGGCGATAAGTC
GTGTCTTACCGGGTTGGACTCAAGACGATAGTTACCGGATAAGGCGCAGCGGTCGGGCTGAACGGGGGGTTCGTGCACACAGCCCA
GCTTGGAGCGAACGACCTACACCGAACTGAGATACCTACAGCGTGAGCTATGAGAAAGCGCCACGCTTCCCGAAGGGAGAAAGGCG
GACAGGTATCCGGTAAGCGGCAGGGTCGGAACAGGAGAGCGCACGAGGGAGCTTCCAGGGGGAAACGCCTGGTATCTTTATAGTCC
TGTCGGGTTTCGCCACCTCTGACTTGAGCGTCGATTTTTGTGATGCTCGTCAGGGGGGCGGAGCCTATGGAAAAACGCCAGCAACG
CGGCCTTTTTACGGTTCCTGGCCTTTTGCTGGCCTTTTGCTCACATGTTCTTTCCTGCGTTATCCCCTGATTCTGTGGATAACCGT
ATTACCGCCTTTGAGTGAGCTGATACCGCTCGCCGCAGCCGAACGACCGAGCGCAGCGAGTCAGTGAGCGAGGAAGCGGAAGAGCG
CCCAATACGCAAACCGCCTCTCCCCGCGCGTTGGCCGATTCATTAATGCAGCTGGCACGACAGGTTTCCCGACTGGAAAGCGGGCA
GTGAGCGCAACGCAATTAATGTGAGTTAGCTCACTCATTAGGCTTATTTGTACAGCTCATCCATGCCACCGGTAGAATGACGACCC
TCCGCGCGCTCATATTGCTCTACGATCGTATAATCTTCATTATGAGAGGTGATGTCCAGTTTAATATTCACATTGTACGCGCCAGG
CAGCTGCACAGGTTTCTTGGCTTTGTACGTGGTTTTCACTTCAGCGTCATAATGGCCGCCATCTTTCAGTTTCAGGCGCTGTTTAA
TTTCGCCTTTCAGCGCACCATCTTCCGGATACATGCGTTCGCTAGACGCCTCCCAACCCATCGTCTTTTTCTGCATCACCGGGCCA
TCAGATGGAAAATTAGTACCACGCAGTTTAACTTTATAGATGAACTCGCCATCCTGCAGGGAGGAGTCCTGAGTGACGGTCACGAC
ACCACCATCTTCAAAATTCATTACGCGTTCCCACTTGAAACCTTCCGGAAAAGACAGCTTCAGATAGTCCGGGATATCCGCTGGGT
GTTTAACATACGCTTTAGAACCGTACATAAATTGCGGGCTCAGGATGTCCCACGCAAAAGGCAGCGGGCCGCCTTTAGTCACTTTC
AGTTTGGCGGTCTGGGTGCCTTCATACGGACGGCCCTCGCCTTCGCCTTCGATCTCGAACTCGTGACCGTTAACAGAACCCTCCAT
GTGAACTTTGAAGCGCATGAACTCTTTAATGATAGCCATGTTATCCTCCTCGCCCTTGGAAACCATATTCACCACCCTGAATTGAC
TCTCTTCCGGGCGCTATCATGCCATACCGCGAAAGGTTTTGCACCATTCGATGGTGTCGGGTACCAAGGAGATGGCGCCCAACAGT
CCCCCGGCCACGGGGCCTGCCACCATACCCACGCCGAAACAAGCGCTCATGAGCCCGAAGTGGCGAGCCCGATCTTCCCCATCGGT
GATGTCGGCGATATAGGCGCCAGCAACCGCACCTGTGGCGCCGGTGATGCCGGCCACGATGCGTCCGGCGTAGAGGATCCGGGCTT
ATCGACTGCACGGTGCACCAATGCTTCTGGCGTCAGGCAGCCATCGGAAGCTGTGGTATGGCTGTGCAGGTCGTAAATCACTGCAT
AATTCGTGTCGCTCAAGGCGCACTCCCGTTCTGGATAATGTTTTTTGCGCCGACATCATAACGGTTCTGGCAAATATTCTGAAATG
AGCTG***TTGACA****ATTAATCATCGGCTCG****TATAAT****GTGTGG****A**ATTGTGAGCGGATAACAATT*TCACACAGGAAACA**TGACCTAAGGAG**
GTAAATAATGCGTAAAGGCGAAGAGCTGTTCACTGGTTTCGTCACTATTCTGGTGGAACTGGATGGTGATGTCAACGGTCATAAGT
TTTCCGTGCGTGGCGAGGGTGAAGGTGACGCAACTAATGGTAAACTGACGCTGAAGTTCATCTGTACTACTGGTAAACTGCCGGTA
CCTTGGCCGACTCTGGTAACGACGCTGACTTATGGTGTTCAGTGCTTTGCTCGTTATCCGGACCACATGAAGCAGCATGACTTCTT
CAAGTCCGCCATGCCGGAAGGCTATGTGCAGGAACGCACGATTTCCTTTAAGGATGACGGCACGTACAAAACGCGTGCGGAAGTGA
AATTTGAAGGCGATACCCTGGTAAACCGCATTGAGCTGAAAGGCATTGACTTTAAAGAAGACGGCAATATCCTGGGCCATAAGCTG
GAATACAATTTTAACAGCCACAATGTTTACATCACCGCCGATAAACAAAAAAATGGCATTAAAGCGAATTTTAAAATTCGCCACAA
CGTGGAGGATGGCAGCGTGCAGCTGGCTGATCACTACCAGCAAAACACTCCAATCGGTGATGGTCCTGTTCTGCTGCCAGACAATC
ACTATCTGAGCACGCAAAGCGTTCTGTCTAAAGATCCGAACGAGAAACGCGATCACATGGTTCTGCTGGAGTTCGTAACCGCAGCG
GGCATCACGCATGGTATGGATGAACTGTACAAATAA

Validation of FACS enrichment system for chimeric TFs

Construction of control strains

The *E. coli* K-12 gene encoding LacI (NCBI Accession AAC73448) was PCR amplified using primers K-12 LacI-P.I-RBS 5’ / K-12 LacI-P.IIB 3’ (Supplementary Data 2), which added 30 bp of homologous sequence to pCKT (Supplementary Data 1) polylinker on both ends of the *lacI* PCR product (also adding a consensus RBS to the 5′-end of the gene between the homology arm and the starting

codon). These homology arms allowed the directional cloning through Gibson assembly into a SmaI (NEB, Ipswich, MA) digested pCKT plasmid. The assembled product was named pCKTRBS-LacIwt and transformed into chemically competent NEB5-alpha cells (NEB, Ipswich, MA). The reporter vector pHC_DYOLacI-R was electroporated into the strain expressing LacI giving place to NEB5-alpha (pCKTRBS-LacIwt, pHC_DYOLacI-R). The name of this final strain was abbreviated to AYC wtLacI (Supplementary Data 1).

The control chimera $SLCP_{GL}$[5] has been identified along this paper as LacI-GGBP-OD to simplify its name and standardize it to the nomenclature of chimeras mentioned in this work. However, it should technically be called LacI-J6-GGBP-OD, as J6 LNK (Supplementary Table 2) was included in its structure. To assemble the chimera, the OD region of LacI was obtained as a synthetic dsDNA fragment flanked by restriction sites for SmaI and XbaI (IDT, Coralville, CA). The SmaI/XbaI (NEB, Ipswich, MA) digested OD fragment was cloned into a pCK01 (Supplementary Data 1) plasmid digested with the same restriction enzymes. The resulting pCK-[OD] plasmid was linearized with SmaI. DBD-LacI and GGBP were PCR amplified using the primer pairs K-12 LacI-P.I-RBS 5' / K-12 LacI-L 3' and K-12 GGBP(mglB)-L 5' / K-12 GGBP(mglB)-OD 3' (Supplementary Data 2), respectively. The amplified DBD-LacI was flanked in its 5´-end by a 30 bp homology arm to the region upstream of SmaI in pCK01. On the other hand, GGBP was flanked by a 30 bp long arm homologous to OD. Both DBD-LacI and GGBP were flanked in their 3´ and 5´-ends, respectively, by the J6 sequence. DBD-LacI and GGBP were Gibson assembled into SmaI linearized pCK-[OD] forming the intermediate vector pCKQ_LacI-GGBP-OD_G. In parallel, the plasmid pCKT, a pCK01 chassis modified to replace *Plac* by a *tetR-PtetO* cassette as shown in Supplementary Fig. 2, was constructed and OD cloned in it as a SmaI/XbaI fragment (see above) to originate pCKT-[OD]. An entire *lacI-J6-GGBP* fragment was PCR amplified from pCKQ_LacI-GGBP-OD_G using the oligonucleotide pair K-12 LacI-P.I-RBS 5' / K-12 GGBP(mglB)-OD 3' (Supplementary Data 2) and Gibson assembled into a SmaI linearized pCKT-[OD], forming to pCKTRBS-LacI-GGBP-OD. The final sequence of the chimera known as LacI-GGBP-OD is shown below in this Supplementary Methods where it is compared to the original $SLCP_{GL}$[5]. NEB5-alpha cells

(NEB, Ipswich, MA) were initially transformed with pCKTRBS-LacI-GGBP-OD and subsequently electroporated with pHC_DYOLacI-R. The resulting NEB5-alpha (pCKTRBS-LacI-GGBP-OD, pHC_DYOLacI-R) strain was renamed AYC ChTFGlc.

Wild-type TF control: LacI

The initial validation of the screening process was performed using as a control the wild-type transcriptional repressor LacI[24]. We used the AYC wtLacI strain (Supplementary Data 1) to assay the FACS enrichment of cells expressing LacI from pCKTRBS and carrying pHC_DYOLacI-R as reporter plasmid. The construction of the strain is detailed in Methods. AYC wtLacI cells were grown in media supplemented either with aTc exclusively or with aTc and IPTG. The cultures containing aTc showed reduced levels of GFP associated fluorescence (LacI expressed, *Ptac*-GFP repressed), whereas the cultures in which IPTG was added (LacI expressed and induced, *Ptac*-GFP de-repressed) showed GFP levels comparable to control NEB5-alpha (pHC_DYOLacI-R) cells. This assay demonstrated the functionality of the LacI-*Ptac*-GFP system in AYC wtLacI. A culture of AYC wtLacI supplemented with aTc and IPTG was mixed 50:50 with a subset of AYC Lib-Ch-OD supplemented with aTc. The culture mix underwent Positive Sorting as described above. The proportion of pCKTRBS-OD-*Chimera* and pCKTRBS-LacIwt was evaluated pre- and post-sorting by Sanger sequencing. It showed the proportion of pCKTRBS-LacIwt in the population had increased from 50.4 ± 2.5% to 94.4 ± 2.7%, confirming the viability of the enrichment system for wild-type TFs.

Previously-characterized chimeric TF control: LacI-GGBP-OD ($SLCP_{GL}$)

Despite the validation of the screening with LacI there were concerns about the effectiveness of the screening when applied to chimeric repressors. DBDs could lose DNA affinity when integrated into chimeric TFs. LacI-GGBP-OD was a clear example of this problem, as it had been reported to bind DNA with lower affinity than LacI[5]. This diminished interaction of DBD-LacI with its operators could be due to LacI-GGBP-OD forming a more unstable dimer due to the alteration of the contact area between two

LacI-GGBP-OD molecules all along the LBD. However, since LacI-GGBP-OD is (to the best of our knowledge) the only published chimera whose LBD was a PBP, it was still used as a positive control for our enrichment experiments. LacI-GGBP-OD was cloned into pCKTRBS preserving the domain outline[Citation error] where possible (below). The AYC ChTFGlc strain (Supplementary Data 1) expresses LacI-GGBP-OD from a pCKTRBS chassis and carries the reporter pHC_DYOLacI-R. The construction of this strain is detailed in Methods.

AYC ChTFGlc cells were grown in either LB containing aTc or LB supplemented with aTc and glucose. The cultures containing aTc showed reduced GFP-associated fluorescence (LacI-GGBP-OD expressed, *Ptac*-GFP repressed), whereas the cultures in which glucose was added (LaI-GGBP-OD expressed and induced, *Ptac*-GFP de-repressed) showed GFP levels approximate to basal expression of *Ptac*-GFP in the repressor free strain NEB5-alpha (pHC_DYOLacI-R) (data not shown). These results suggested the inducibility of the LacI-GGBP-OD chimera in the presence of glucose when assayed with the reporter system presented in this work. Subsequently, a culture of AYC ChTFGlc plus aTc and glucose was mixed with a culture of AYC wtLacI plus aTc in different proportions. The mix was subsequently enriched for the cells showing the highest levels of GFP associated fluorescence during FACS (Methods). Individual clones were isolated from the pre- and post-sorted populations and the TFs they carried in pCKTRBS were identified by Sanger sequencing. The distribution of LacI-GGBP-OD and LacI in the pre- and post-sorting populations showed a maximum enrichment of 95.8% in the strain carrying LacI-GGBP-OD, with an average of 88.76±5.51%. These experiments demonstrated the viability of LacI-GGBP-OD as a positive control for the FACS enrichment used in this paper.

The next step consisted on recovering LacI-GGBP-OD from a complex library of chimeric TFs in which it would be present at a very low concentration. These conditions would mimic more closely the actual enrichment of a benzoate-responsive TF. AYC ChTFGlc cells grown in the presence of aTc were added to a culture of AYC Lib-Ch-END cultivated under the same conditions. Bacteria carrying LacI-GGBP-OD represented approximately a 5% of the mixed population. Each chimera present in the culture was expressed due to the presence of aTc. Under these circumstances the expression of GFP from the

reporter promoter, included in the pHC_DYO*DBD*-R, was repressed when a pairing formed between the DBD of chimera and the synthetic operator box in the modified *Ptac*. A Negative Sorting was performed: all non-fluorescent cells, plus the 10% of fluorescent cells with the lower GFP fluorescence, were recovered. Sorted bacteria were grown in LB plus aTc and glucose (repressors expressed, promoters regulated by glucose-sensing chimeras de-repressed). Prior to sorting, a marginal percentage of the population was fluorescent in the emission range of GFP. A sample enriched in the most fluorescent cells was obtained using FACS. The recovered cells were grown again in the presence of aTc and glucose. After this recovery the proportion of GFP fluorescent cells had grown. The top 10% of GFP-positive bacteria were recovered for the next round. In subsequent iterations under the same conditions, the top 5% and 1% (twice, sequentially) were recovered. The composition of the starting library and the one obtained after the 5 cycles of enrichment was analyzed by high-throughput sequencing of the chimeras present in the population (Methods). LacI-GGBP-OD proportion had grown from 4.78% in the original population to 63.72% after sorting. This enrichment demonstrated that a FACS-based screening process could be utilized to recover a functional chimera from a complex library of similar constructs.

Next, we recovered only bacteria expressing the native LacI-GGBP-OD present in AYC Lib-Ch-OD. Here, no extra AYC ChTFGlc cells were supplemented to AYC Lib-Ch-OD. All the elements necessary to reconstruct LacI-GGBP-OD were included in the libraries as an internal positive control. The same experimental design described above for the selection of the exogenous LacI-GGBP-OD was implemented for the enrichment on the endogenous version of the control chimera. Firstly, a Negative Sorting was performed, recovering all the non-fluorescent cells plus the 10% of fluorescent cells with a lower fluorescence. The recovered bacteria were grown in culture media plus aTc and glucose (chimeras expressed, LacI-GGBP-OD regulated promoters de-repressed). 5% of the population showed GFP-associated fluorescence in FACS, and the 50% with the strongest fluorescence levels was recovered for the next round. The cells from this Positive Sorting were grown again in the presence of aTc and glucose. After the first enrichment the proportion of fluorescent cells had grown. In this case the top 10% of GFP-positive cells was recovered. The cycle was repeated recovering, sequentially, the top 5% and top 1% of

GFP-positive bacteria. The composition of the starting library and the one recovered after the 4 cycles of enrichment was analyzed by next gen sequencing of the chimeras present in the population (Methods). LacI-GGBP-OD was undetectable in the starting library (<0.0015% of the population) but represented a 0.25% of the total in the enriched population. An independent replica of the whole process, starting with a fresh Negative Sorting followed by 5 iterative Positive Sortings retrieved a maximum abundance of LacI-GGBP-OD *versus* all the TFs present in the enriched cells of 25.3%. These results showed that a detectable enrichment in a functional chimeric regulator present in very low concentration in our starting libraries could be achieved. Moreover, it also showed that despite the iterative selection process there still was considerable noise in the enrichments, likely due to a certain percentage of incorrect DBD/reporter pairs being carried over the different Positive Selections of the enrichment process.

The inclusion of LacI-GGBP-OD as a built-in positive control included into the assembly of the library was designed so that GGBP was treated as one more of the LBD. All oligonucleotides necessary for the construction of GGBP-based chimeras were included. 285 different "core chimeras" could be glucose-responsive (9044 total different constructions). The overall population of GGBP bearing chimeras in the starting library AYC Lib-Ch-OD should have been close to 6.67% of the total (4522 out of 67830) but was instead 0.09%, increasing to 32.6% after the enrichment process. These data suggest it is possible to find a better glucose-responsive TF (in terms of dynamic range or inducibility) among non-LacI-GGBP-OD chimeras. Glucose biosensors were not the focus of this publication but in the future, we plan to explore a selection of those glucose-sensing TFs in depth. This observation highlights the strength of the chimeragenesis system presented in this work.

Comparison between glucose-recognizing chimeras

Construction name: LacI-GGBP-OD
Origin: This work.
Components of LacI-GGBP-OD chimera:

- DBD-LacI [1-198]
- **J6 LNK** [199-216]
- GGBP [214-1209]

- LacI OD [1210-1320]

>LacI-GGBP-OD (LacI-J6-GGBP-OD)

ATGAAACCAGTAACGTTATACGATGTCGCAGAGTATGCCGGTGTCTCTTATCAGACCGTTTCCCGCGTGGTGAACCAGGCCAGCCA
CGTTTCTGCGAAAACGCGGGAAAAAGTGGAAGCGGCGATGGCGGAGCTGAATTACATTCCCAACCGCGTGGCACAACAACTGGCGG
GCAAACAGTCGTTGCTGATTGGCGTT**GGCTTAATGGGCTTA**ATGAATAAGAAGGTGTTAACCCTGTCTGCTGTGATGGCCAGCATG
TTATTCGGTGCCGCTGCACACGCTGCTGATACTCGCATTGGTGTAACAATCTATAAGTACGACGATAACTTTATGTCTGTAGTGCG
CAAGGCTATTGAGCAAGATGCGAAAGCCGCGCCAGATGTTCAGCTGCTGATGAATGATTCTCAGAATGACCAGTCCAAGCAGAACG
ATCAGATCGACGTATTGCTGGCGAAAGGGGTGAAGGCACTGGCAATCAACCTGGTTGACCCGGCAGCTGCGGGTACGGTGATTGAG
AAAGCGCGTGGGCAAAACGTGCCGGTGGTTTTCTTCAACAAAGAACCGTCTCGTAAGGCGCTGGATAGCTACGACAAAGCCTACTA
CGTTGGCACTGACTCCAAAGAGTCCGGCATTATTCAAGGCGATTTGATTGCTAAACACTGGGCGGCGAATCAGGGTTGGGATCTGA
ACAAAGACGGTCAGATTCAGTTCGTACTGCTGAAAGGTGAACCGGGCCATCCGGATGCAGAAGCACGTACCACTTACGTGATTAAA
GAATTGAACGATAAAGGCATCAAAACTGAACAGTTACAGTTAGATACCGCAATGTGGGACACCGCTCAGGCGAAAGATAAGATGGA
CGCCTGGCTGTCTGGCCCGAACGCCAACAAAATCGAAGTGGTTATCGCCAACAACGATGCGATGGCAATGGGCGCGGTTGAAGCGC
TGAAAGCACACAACAAGTCCAGCATTCCGGTGTTTGGCGTCGATGCGCTGCCAGAAGCGCTGGCGCTGGTGAAATCCGGTGCACTG
GCGGGCACCGTACTGAACGATGCTAACAACCAGGCGAAAGCGACCTTTGATCTGGCGAAAAACCTGGCCGATGGTAAAGGTGCGGC
TGATGGCACCAACTGGAAAATCGACAACAAAGTGGTCCGCGTACCTTATGTTGGCGTAGATAAAGACAACCTGGCTGAATTCAGCA
AGAAAAAAAGAAAAACCACCCTGGCGCCCAATACGCAAACCGCCTCTCCCCGCGCGTTGGCCGATTCATTAATGCAGCTGGCACGA
CAGGTTTCCCGACTGGAAAGCGGGCAGTGA

Construction name: $SLCP_{GL}$

Origin: [5].

Components of LacI-GGBP-OD chimera:

- DBD-LacI [1-198]
- BamHI [199-205]
- GGBP [206-1071]
- SalI [1702-1707]
- LacI OD [11708-1185]

> $SLCP_{GL}$

ATGAAACCAGTAACGTTATACGATGTCGCAGAGTATGCCGGTGTCTCTTATCAGACCGTTTCCCGCGTGGTGAACCAGGCCAGCCA
CGTTTCTGCGAAAACGCGGGAAAAAGTGGAAGCGGCGATGGCGGAGCTGAATTACATTCCCAACCGCGTGGCACAACAACTGGCGG
GCAAACAGTCGTTGCTGATTGGCGTTGGATCCATCTATAAGTACGACGATAACTTTATGTCTGTAGTGCGCAAGGCTATTGAGCAA
GATGCGAAAGCCGCGCCAGATGTTCAGCTGCTGATGAATGATTCTCAGAATGACCAGTCCAAGCAGAACGATCAGATCGACGTATT
GCTGGCGAAAGGGGTGAAGGCACTGGCAATCAACCTGGTTGACCCGGCAGCTGCGGGTACGGTGATTGAGAAAGCGCGTGGGCAAA
ACGTGCCGGTGGTTTTCTTCAACAAAGAACCGTCTCGTAAGGCGCTGGATAGCTACGACAAAGCCTACTACGTTGGCACTGACTCC
AAAGAGTCCGGCATTATTCAAGGCGATTTGATTGCTAAACACTGGGCGGCGAATCAGGGTTGGGATCTGAACAAAGACGGTCAGAT
TCAGTTCGTACTGCTGAAAGGTGAACCGGGCCATCCGGATGCAGAAGCACGTACCACTTACGTGATTAAAGAATTGAACGATAAAG
GCATCAAAACTGAACAGTTACAGTTAGATACCGCAATGTGGGACACCGCTCAGGCGAAAGATAAGATGGACGCCTGGCTGTCTGGC
CCGAACGCCAACAAAATCGAAGTGGTTATCGCCAACAACGATGCGATGGCAATGGGCGCGGTTGAAGCGCTGAAAGCACACAACAA
GTCCAGCATTCCGGTGTTTGGCGTCGATGCGCTGCCAGAAGCGCTGGCGCTGGTGAAATCCGGTGCACTGGCGGGCACCGTACTGA
ACGATGCTAACAACCAGGCGAAAGCGACCTTTGATCTGGCGAAAAACCTGGCCGATGGTAAAGGTGCGGCTGATGGCACCAACTGG
AAAATCGACAACAAAGTGGTCCGCGTACCTTATGTTGGCGTCGACAGAAAAACCACCCTGGCGCCCAATACGCAAACCGCCTCTCC
CCGCGCGTTGGCCGATTCATTAATGCAGCTGGCACGACAGGTTTCCCGACTGGAAAGCGGGCAGTGA

Benzoate-binding chimeras

Addgene ID: #102925
Chimeric TF publication name: ChTFBz01
Chimeric TF systematic name: CbnR-ABE44898-OD
Chimeric TF assembly name: cbnR_88_Nt_core-Nolink-ABE44898-OD

Construction components:

- DBD-CbnR (core) [1-264]
- ABE44898 [265-1449]
- LacI OD [1450-1557]

>ChTFBz01

ATGGAATTCCGGCAGCTCAAGTATTTCATCGCCGTCGCGGAAGCAGGGAACATGGCTGCAGCAGCCAAGCGCCTGCACGTCTCACA
GCCCCCCATCACGCGGCAGATGCAAGCCCTGGAAGCGGACTTGGGCGTCGTGCTTCTGGAGCGAAGCCACCGCGGGATCGAACTCA
CCGCCGCAGGTCACGCTTTCCTGGAGGATGCGCGCCGCATCCTGGAGTTGGCAGGCCGTTCGGGTGATCGCTCCCGCGCCGCCGCG
CGGGGCATGACCTCACGCCGACTTGTTTTGACCCGCAGTGCTGCCGTTATCGGCGCCGCATCCACTGGCCTGCTGCTGCCCGAAAT
CGTGCGCGCACAGTCGGGCAAGGTGCGTGTGGGCTTCATGTTGCCCTACACCGGCACCTTTGCCCAACTCGGGGTGGCCATTGAAA
ACGGCTTTCGCCTGGCGATCAATGAGCAAGGCGGCAAGCTGGGCGGCCGCGAGATCGAATACTTCAAGGTCGACGATGAGTCCGAG
CCGTCCAAAGGCATTGAAAACGCCAGCAAGCTGGTGCAACGCGACAAGGTCGATGTACTGGTCGGCACGGTGCACTCCGGTGTGCA
GATGGGTATCCAGAAGGTCGCGCGCGACAGCGGCGTACTGAGCCTGATTCCCAACGCGGGCGTGCATGCCGCCACCCGCAGCCTGT
GCGCACCCAATGTGTTCCGTACCTCCTTCAGCAACTCGCAACCTACCCGCGCCCTGGGTCAGGCCATGATCGCAAAAGGCCATAAA
AAAGCCGTCTGGATCACCTGGAAATATGCAGCCGGCGACGAGGCCTTTGAAGGCTTCAAGGAAAGCTACACAGCGGCCGGCGGCAC
CATTATCAAGGAACTGGGCCTGCCCTTCCCCAACGTTGAATTCCAGGCGCTGCTGACCGAAATCGCCGCACTCAAGCCCGATGCCG
TGGCCTGCTTCTTCGCCGGCGGCGGCGCCGCGAAGTTCATCCGCGACTACGCAGCGGCGGGCCTCAAAGGCAAGATCCCGCTCTAC
GGTTCCGGCTTCCTGACCGAAGGCGTGCTGGACGCGGCCGGCCCTGCGGCGGACGGCATCATCACCACCATGCACTACAGCGACTC
GCTGGACACGCCGCGCAACAAGAAATTCCGCCTCGACTACGCCAAAGCCTTCAAGACGCAACCCGATGTGTACGCGGTGCAGGGCT
ATGACACCGGCCTGCTGCTGATCCAGGGCGCCAACGCCGTCAAGGGCGACCTGGCCAACAAGCCCGCGCTCTACAAGGCCATGGAA
GGCGCCACCATCGACAGCCCGCGCGGCAAGTGGACCATGAGCAAGGCACACAACCCGGTGCAGGACATGTACCTGCGTGTGGTGGA
AAACAAGGAAAACAAGGTGCTCGGCGTTGCGGCCAAGGCACTGGCCGACTCCGGCGCCGGCTGCAAGATGGGCAAAAGAAAAACCA
CCCTGGCGCCCAATACGCAAACCGCCTCTCCCCGCGCGTTGGCCGATTCATTAATGCAGCTGGCACGACAGGTTTCCCGACTGGAA
AGCGGGCAGTGA

Addgene ID: #102926
Chimeric TF publication name: ChTFBz02
Chimeric TF systematic name: LmrR-BzdB1_nSP
Chimeric TF assembly name: lmrR_81_Nt_core-Nolink-bzdB1_nSP_optimized
Construction components:

- DBD-LmrR (core) [1-243]
- BzdB1_nSP [244-1341]

>ChTFBz02

ATGGCAGAAATACCAAAAGAAATGTTACGAGCCCAAACCAATGTAATTTTGCTCAATGTCCTAAAACAAGGAGATAATTATGTTTA
CGGTATTATCAAACAAGTCAAGGAAGCCTCGAATGGAGAAATGGAACTTAATGAAGCCACGCTCTATACGATCTTTAAAAGACTTG
AAAAGGATGGGATTATCAGTTCTTATTGGGGAGATGAAAGTCAAGGCGGGCGTCGCAAGTATTACCGACTGCAGAGCGCAAATAAA
ATTCGTGTGGGTTTAATGCTGCCTTACACCGGTACCTATGCGAGCCTGGGTAATGCCATTACCAATGGCTTTAAACTGGCCGTTGA
ACAAGGTGGTGGTAAACTGGGTGGTCGCGAGATTGAGTACTTCACCGTGGATGATGAAAGCGATGCCGCAAAAGCGCCGGAAAATG
CCAACAAACTGATTAAACGTGATAATGTGGATGTGCTGATTGGCACCGTTCATAGCGGCGTTGCACTGGCAATGACCCGTGTTGCA
CGTGAAACCAAAACACTGCTGATCGTTCCGAATGCGGGTGCAGATGAAATTACCGGTCCGCTGTGTAGCCCGAATATTTTTCGTAC
AAGCTTTAGCGCATGGCAGCCGGCATATGCAATGGGTCAGGTGATGGCCGAAAAAAAACATAAAAATGTGGTGACCCTGAGCTGGA
AATATAGCTTCGGCGAACAGAGCGTGGCAGGTTTCAAGGAAGCCTTTGAAAAAGGCGGCGGCAAAGTGGTTAAAGAAATGTATCTG
CCGTTTCCGAACGTGGAATTTCAGCCGTATCTGACGGAAATTGCCGCACTGAAACCGGATGCCGTGTTTGTGTTTTTTGCCGGCGG
TGGTGCAGTGAAATTTGTGAAAGATTATGACGCCGCAGGTCTGAAAAAAACCATTCCGCTGTATGGTAGCGGTTTTCTGACCGATG
GTACCCTGGAAGCACAGGGTGATAGCGCAGAAGGTATTCTGACCACCCTGCATTATGCAGATGGTCTGGATATTGCAAAAGATAAA
GCCTTTCGTACCGCCTATGCCATGGCCTTCAAAACCCAGCCGGATGTTTATAGCGTTCAGGGTTATGATGCAGCACAGCTTTTTGC
AGCAGGTTTAGCGGGTGTTGCCGGTGATGTTACCAAACGTGAAGCACTGATTGCAGCAATGGAAAAAGCAACCATTGATAGCCCGC
GTGGTCGTTTTACCCTGAGTAAAGCACATAATCCGGTGCAGGATATTTACCTGCGGAAAGTGGAAGCCAAACAGAATAAAGTGATC
GCAGTGGCAGCAAAAGCCCTGGCAGATCCGGCACGTGGTTGTCGTATGTAA

Supplementary Table 1. DNA-binding Domains (DBD) used in this study.

NAME	FAMILY	CRYSTAL	ASS. LEVEL ON DNA	ACC.	NATIVE INDUCER	"CORE" DBD DOMAIN (aa)	"END" DBD DOMAIN (aa)	COMMENTS ON DOMAIN SELECTION	[3D CONFIGURATION BDB]-[LNK]
ArgR	ArgR	3ERE	Dimer of trimers	AAK45964	Arginine	1-80	81, 82, 83, 84, 85, 86, 87, 88	Based on 3D structure 1-88. Ref. for the family:[25]	[HTH]-2[short B]
FL11	AsnC/Lrp	2ZNY	6 dimers	BAA30629	Lysine Arginine	1-58	59, 60, 61, 62, 63, 64, 65, 66	Based on 3D structure 1-66. Ref. for the family:[26]	[HTH]-[short B]
DeoR	DeoR	-	Octamer?	AAC73927	Phosphorylated sugar	1-47	62, 63, 64, 65, 66, 67, 68, 69, 70, 71, 72, 73, 74, 75	Based on 3D models (Swissprot and Phyre2).	[HTH] (Model)
LacI	LacI/GalR	1LBG (DNA)	Dimer (Tetramer)	AAC73448	Lactose	1-61	62, 63, 64, 65, 66	Based on[5, 27].	[HTH]
TreR	LacI/GalR	1BYK (Ct)	Dimer	Q2M666	Trehalose-P	1-62	63, 64, 65, 66, 67	Based on[27] and comparison with LacI	[HTH] (LacI/GalR family)
FadR	GntR	1H9T	Dimer	AAC74271	Acyl-CoA	1-73	74, 75, 76, 77, 89, 90, 91, 92, 93	Based on 3D structure 89, 90, 91, 92, 93. Based on[28] 1-77.	[HTH]-2[short H]
TtgV	IclR	2XRO 2XRN	Tetramer	AAK69562	1-naphthol, 4-nitrotoluene	1-71	72, 73, 74, 75, 83, 84, 85, 86, 87	Based on 3D structure 1-75 just include HTH. 83-87 include the subsequent alpha-helix. Ref. for the family:[29]	[HTH]-2[short B]-[short H]
CnbR	LysR	1IXC	Tetramer (dimer of dimers)	BAA74529	*cis,cis*-muconate	1-88	88, 89, 90	Based on 3D structure 1-90. Ref. for the family:[30, 31].	[HTH]-[long H]
CueR	MerR	1Q05	Dimer	AAC73589	Copper	1-103	104, 110, 111, 114	Based on[32].	[HTH]-[long H]
MetJ	MetJ	1MJL, 1MJ2	Dimer	AAA24163	S-adenosylmethionine	1-61	62, 63, 64, 65, 66, 67	Based on[33].	[RHH]
ModE	ModE	1B9M	Dimer	AAB06892	Molybdate	1-105	106, 115, 122, 126	Based on 3D structure. [34] used to avoid the metal binding amino acids.	[HTH]-[long H]-[short H]
LmrR	PadR	3F8C	Dimer	CAL96929	Hoescht	1-81, 1-3+16-80	95	Based on[35]. The domain with the deletion removes amino acids putatively related to the coordination of the native ligand.	[wHTH]-[long H]
TtgR	TetR	2UXI (phloretin)	Dimer	AFO46103	Phloretin, quercetin	1-45	46, 47,48, 81, 82, 83, 84 85	Based on 3D structure. 1-48 just include the HTH domain, 81-85 include an extended alpha helix. Ref. for the family:[20].	[HTH]-[long broken H]
QacR	TetR	1JTX (crystal violet)	Dimer (Tetramer)	ADK23698	Rhodamine	1-45	46, 47, 48, 49, 71, 72, 73, 74, 75	Based on 3D structure. 1-49 just include the HTH domain, 71-75 include an extended alpha helix. Ref. for the family:[20].	[HTH]-[long H]
Xre	Xre	2OFY *Rhodococcus*	?	AAA22894	Unknown	1-56	57, 58, 59, 60, 65, 66, 67, 68, 69, 70	Based on 3D structure. No much information about domain structure.	[HTH]-[short H]

HTH, Helix-Turn-Helix; B, Beta strand; RHH, Ribbon-helix-Helix; wHTH, Winged-HTH

Supplementary Table 2. Linkers (LNK) used in this study.

**Linkers (LNK)**			
**NAME**	**SEQUENCE**	**LENGTH (aa)**	**SOURCE**
BBa_J18920	GS	2	iGEM Parts Registry
BBa_K243004<br>BBa_K648005	GGSG	4	iGEM Parts Registry
BBa_J18921	(GS)3	6	iGEM Parts Registry
BBa_K648007	(GGS)2	6	iGEM Parts Registry
BBa_K243005	(GGSG)2	8	iGEM Parts Registry
BBa_J18922	(GS)5	10	iGEM Parts Registry
BBa_K243006	(GGSG)3	12	iGEM Parts Registry
BBa_K157013	(GGGGS)5	15	iGEM Parts Registry
BBa_K416001	(GGGSG)3	15	iGEM Parts Registry
J6	(GLM)2	6	This work
B4	(EK)2	4	Personal communication (B. Stranges)
B6	(EK)3	6	Personal communication (B. Stranges)
B8	(EK)4	8	Personal communication (B. Stranges)
BBa_K133132	SACYCELS	8	iGEM Parts Registry
BBa_K105012<br>BBa_K648006	GENLYFQSGG	10	iGEM Parts Registry
BBa_K157009	RPACKIPNDLKQKVMNH	17	iGEM Parts Registry
BBa_J176131	(EAAAR)4	20	iGEM Parts Registry
LBzdR	QSPELTLLIQYLSRFPPKTHEWARRLLQNELESSGRSARRQ	41	[36]

Supplementary Table 3. Estimation of the cloning efficiency of the libraries and how cloned TFs were distributed among the different Classes of chimeras.

"CORE CHIMERAS"		*E. coli* (Ch-END)		*E. coli* (Ch-OD)	
	Expected distribution (%)	Distribution (%)	Constructions identified (%) (*)	Distribution (%)	Constructions identified (%) (*)
**Class I**	18.44	20.94	1.57	18.82	1.5
**Class II**	9.38	13.75	1.03	12.94	1.03
**Class III**	44.06	59.69	4.47	60.59	4.82
**Class IV**	4.38	5.62	0.42	7.65	0.61

TOTAL CHIMERAS		*E. coli* (Ch-END)		*E. coli* (Ch-OD)	
	Expected distribution (%)	Distribution (%)	Constructions identified (%) (*)	Distribution (%)	Constructions identified (%) (*)
**Class I**	0.71	63.68	0.36	60.28	0.38
**Class II**	22.11	7.11	0.04	6.15	0.04
**Class III**	43.65	23.95	0.13	26.00	0.16
**Class IV**	33.52	5.26	0.03	7.57	0.05

This is the most conservative estimation of the number of chimeric genes that were assembled in the cloning process and were nontoxic in *E. coli* cells carrying them. As we sought to perform a functional screening of chimeric TFs that could detect glucose (positive control) and benzoate (actual target) the next generation sequencing of the libraries (Methods) did require 100% coverage. The percentage of constructs identified, (*), are assigned based on a coverage of the libraries ranging between 12 and 15%.

Supplementary Table 4. Percentage of cells recovered in every iteration of the positive selections for benzoate-recognizing chimeric TFs.

ITERATION	AYC Lib-Ch-END	AYC Lib-Ch-OD
**1**	100%	100%
**2**	10%	10%
**3**	1%	5%
**4**	1%	1%
**5**	-	1%

Supplementary Figure 1. Abundance of chimeric TFs based on their size.

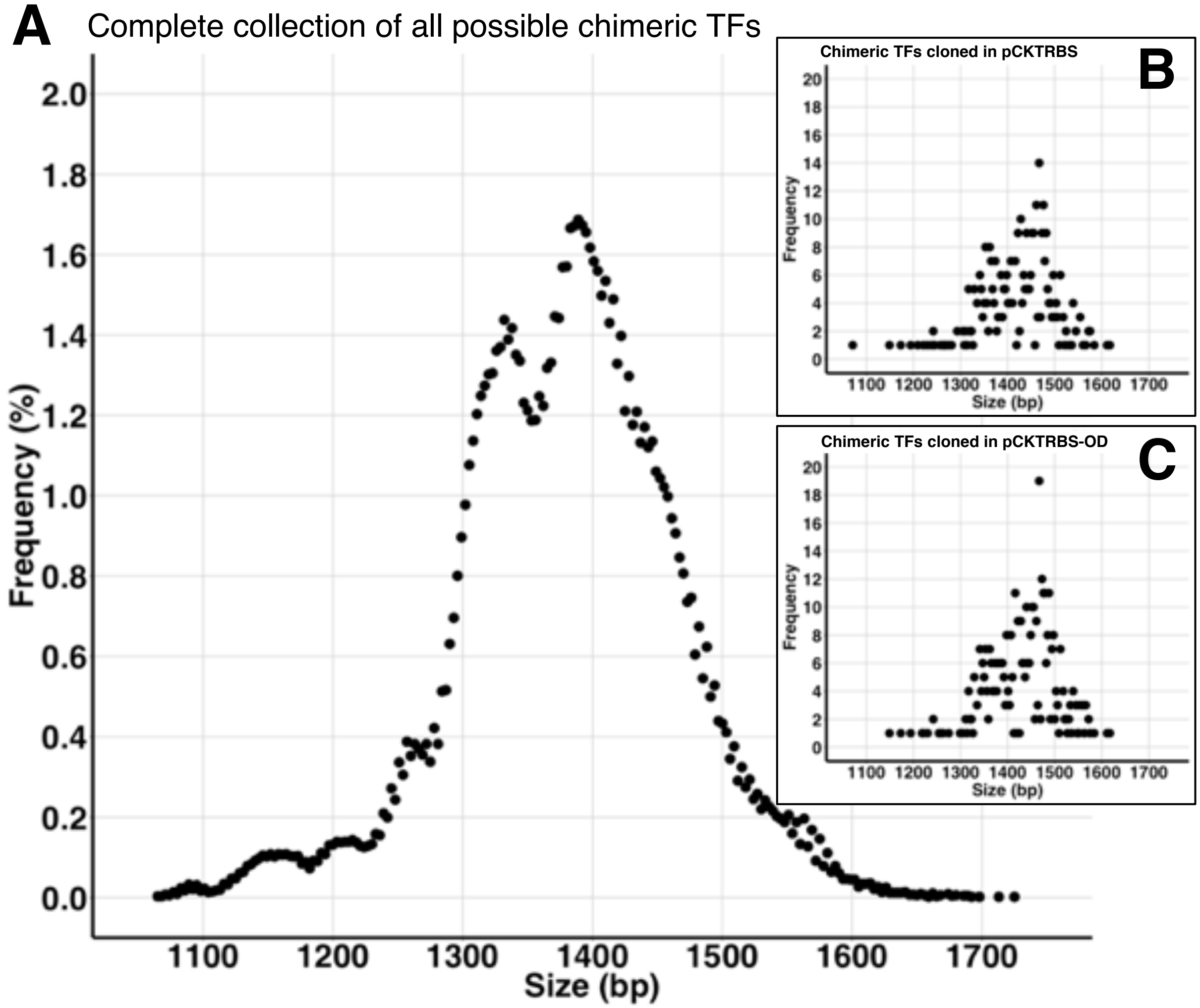

These three plots represent the whole spectrum of the length (bp) of the chimeric genes contained in our TF library and the frequency with which every given gene size appears (as percentage). Panel A represents the distribution for the complete library of fusion genes as it was designed. The smaller panels represent the actual distribution as observed when the library was cloned in pCKTRBS (Panel B) and in pCKTRBS-OD (Panel C). The maximum abundance peaks in the three panels correspond to 1389 bp in Panel A, and 1467 bp in Panels B and C. Data in Panels B and C was obtained by sequencing a subset of the chimeric TFs cloned in *E. coli* (Ch-END) (Panel B) and E. coli (Ch-OD) (Panel C) as described in Methods.

Supplementary Figure 2. GFP expression of *Ptac*-derived synthetic promoters compared to wild-type.

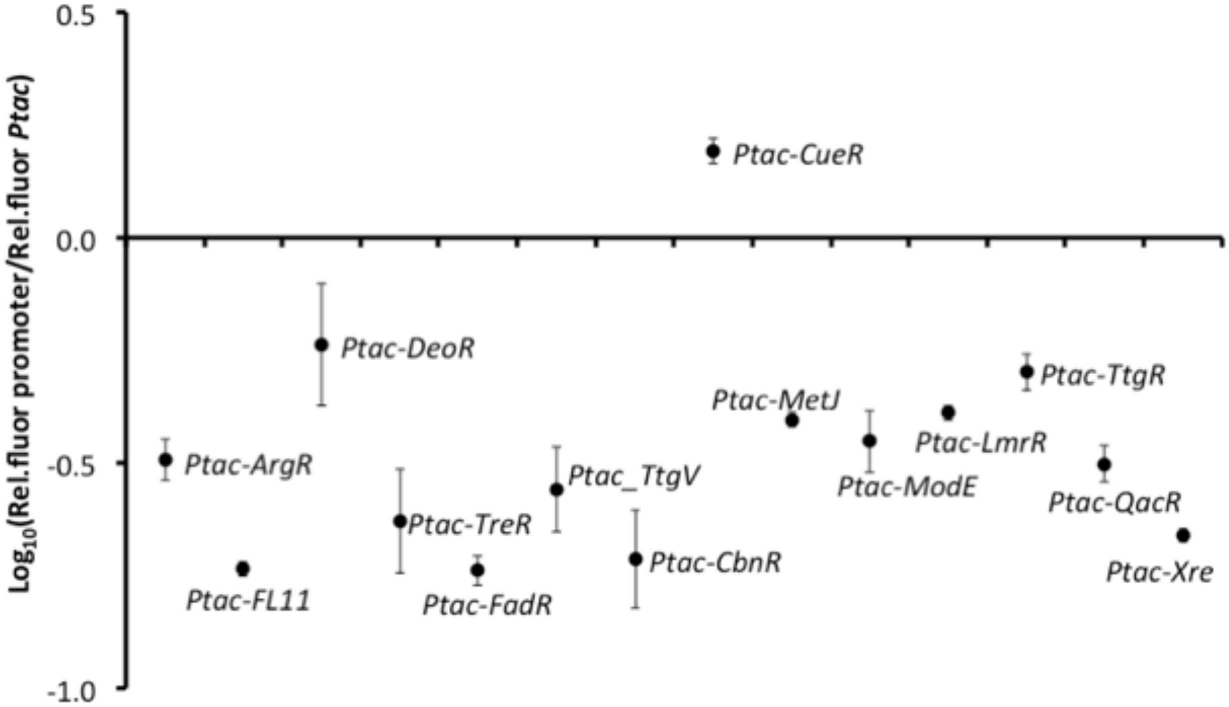

Every dot corresponds to the $\log_{10}$ of the relative expression of a given synthetic promoter (Fluorescence in arbitrary units / OD600) corrected by the relative expression of *Ptac*. Promoters over the X axis showed a stronger relative fluorescence than *Ptac*, whereas promoters below the X axis showed a diminished expression. The promoters were assayed using NEB5-alpha *F′ I*^{q} (pHC_DYO*DBD*-R) strains and their activity compared to that of the reference strain NEB5-alpha *F' I*^{q} (pHC_DYOLacI-R). The relative promoter strength presented here was calculated after 3 hours of growth in the conditions described in Methods (n=3-12). Error bars correspond to Standard Deviation (s.d.).

Supplementary Figure 3. Relative abundance of chimeric TFs after enrichment for benzoate recognition.

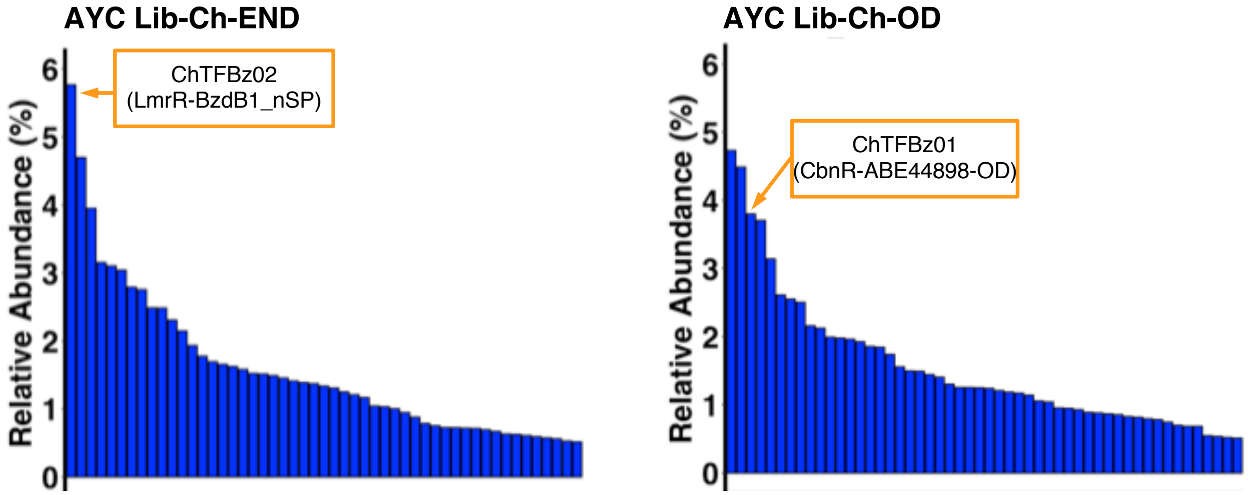

Relative abundance (%) of chimeric genes with an abundance higher than 0.5% in AYC Lib-Ch-END (left panel) and AYC Lib-Ch-OD (right panel) after 4 and 5 enrichment cycles, respectively, for benzoate-recognizing TFs. Every column represents the abundance of a given chimera in the sorted library. Columns corresponding to the constructions ChTFBz01 (CbnR-ABE44898-OD) and ChTFBz02 (LmrR-BzdB1_nSP) characterized in this work are indicated with orange arrows.

Supplementary Figure 4.

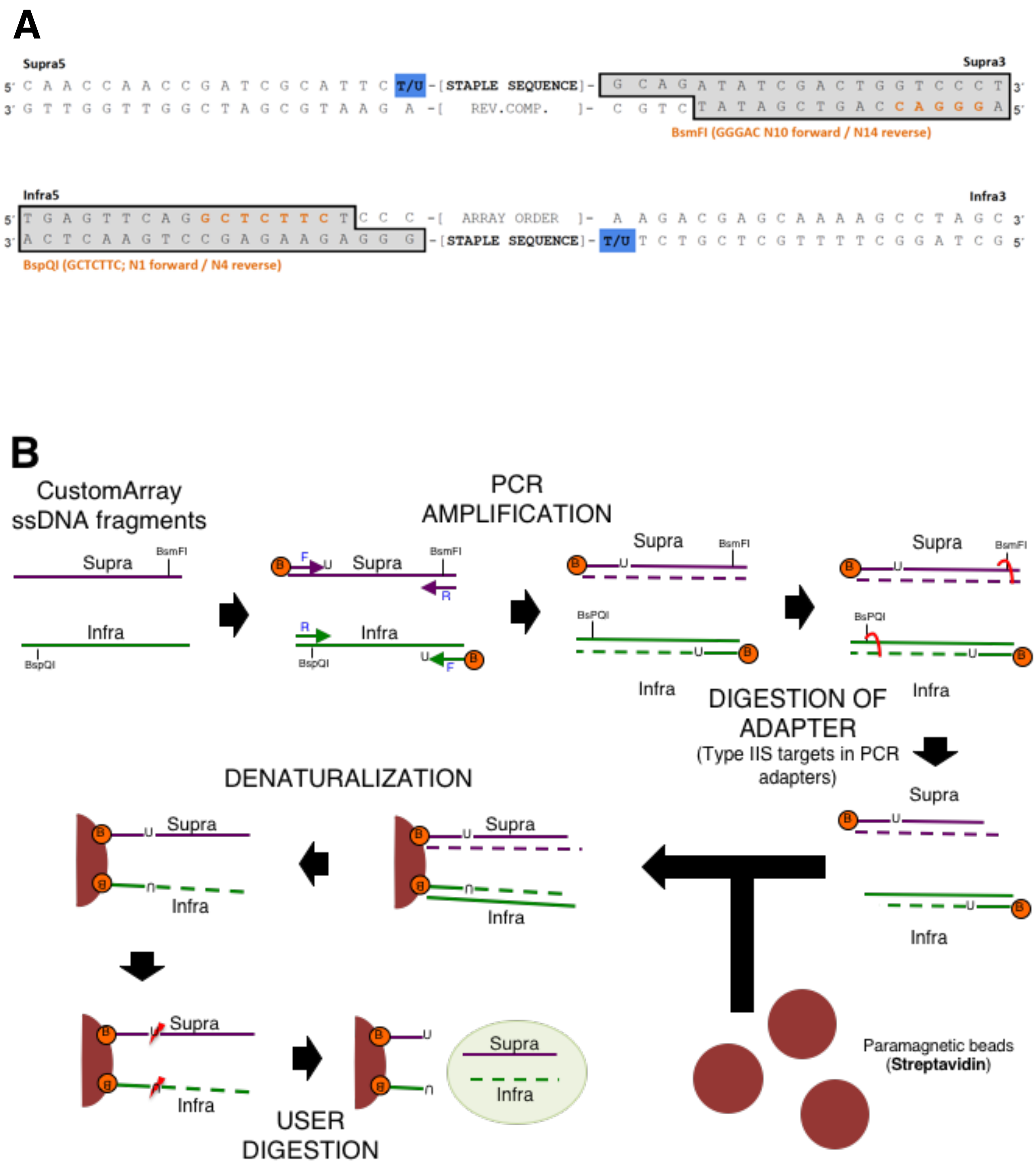

a) Sequences flanking Supra and Infra oligonucleotide staples for eLCR assembly. The staple oligonucleotides of interest would be located in the place indicated between brackets. In the case of the Supra oligonucleotides the staple sequence corresponds with the one included in the CustomArray DNA order. The Supra sequence was ordered flanked in its 5′ side by Supra5 (CAACCAACCGATCGCATTCT) and in its 3′ by Supra3 (GCAGATATCGACTGGTCCCT), that act as adapters for PCR amplification. In the case of the the Infra oligonucleotides the sequence ordered in the CustomArray DNA order was the reverse-complementary of the staple sequence, indicated as "ARRAY ORDER" between brackets, flanked by the PCR adapters Infra5 (TGAGTTCAGGCTCTTCTCCC) and Infra3 (AAGACGAGCAAAAGCCTAGC). The ssDNA fragments Supra5-[Staple sequence]-Supra3 and Infra5-[Array Order]-Infra3 fragments were amplified as dsDNA using the primer pairs BioT-Lib_Adap_supra_F-U / Lib_Adap_supra_R and BioT-Lib_Adap_infra_F-U / Lib_Adap_infra_R, respectively. The resulting dsDNA fragments are shown above and were modified by carrying on the strand where the staple sequence is located: a) U instead of a T immediately upstream of the staple sequence (shadowed in blue); b) a biotin at the 5′ of that same strand. The PCR adapters that did not include the sequence necessary for the introduction of the U incorporated target sequences for two Type IIS restriction enzymes. The areas shadowed in grey correspond with the dsDNA fragment that was removed in the purification process after cleavage by BsmFI (GGGAC) and BspQI (GCTCTTC), both indicated in orange.

b) Amplification process and removal of adapters. CustomArray ssDNA fragments (Supra, solid purple line; Infra, solid green line) were PCR amplified using the primer pairs indicated in A). The reverse-complementary strands of the original ssDNA fragments are indicated as broken lines. After the amplifications one of the adapters was partially removed by digestion with two Type IIS enzymes (BsmFI and BspQI) whose cutting on the dsDNA is represented with short red lines. The preparation was incubated afterwards with paramagnetic beads coated with streptavidin (maroon). The streptavidin of the beads captured dsDNA fragments carrying biotin. The strands that did not contain the staple oligonucleotide were removed by denaturalizing the DNA under high temperature and alkalyne conditions. Finally, the remaining adapter sequence was removed by USER digestion, separating the staple oligonucleotide by cutting at the uracil introduced in the PCR amplification.

Supplementary Figure 5. Main technical features of FACS enrichment.

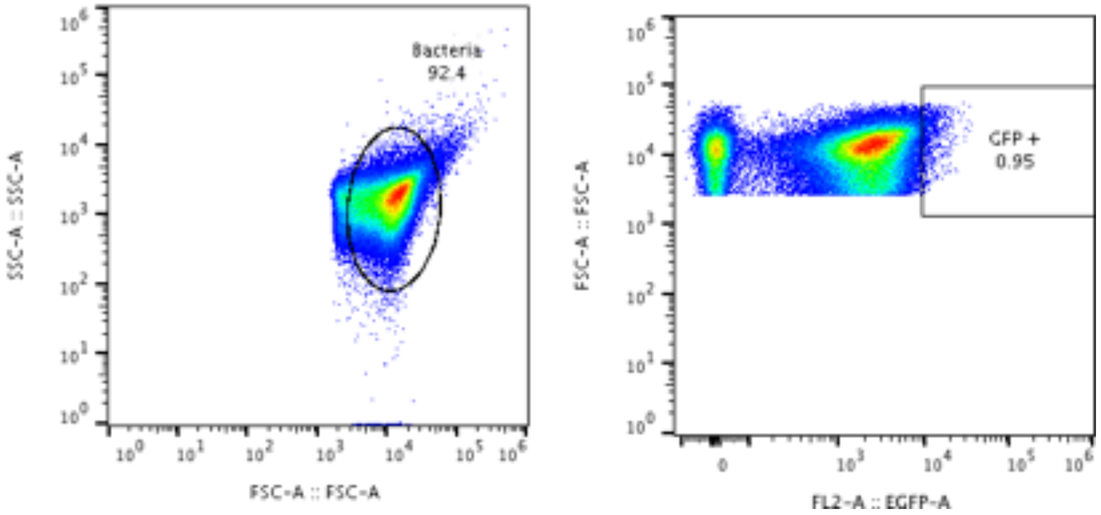

Prototypical gating arrangement: bivariate plot of FSC (forward scatter) area *vs.* BSC (back scatter) area, followed by a rectangular gate containing various percentages of $EGFP^{+}$ (Enhanced Green Fluorescence Protein-Positive) cells on a bivariate plot of FSC area *vs.* EGFP area.

5) Supplementary References

1. Riggs, A. D., Newby, R. F. & Bourgeois, S. lac repressor--operator interaction. II. Effect of galactosides and other ligands. *J. Mol. Biol.* **51,** 303–314 (1970).
2. Raman, S., Taylor, N., Genuth, N., Fields, S. & Church, G. M. Engineering allostery. *Trends Genet.* **30,** 521–528 (2014).
3. Müller-Hartmann, H. & Müller-Hill, B. The side-chain of the amino acid residue in position 110 of the Lac repressor influences its allosteric equilibrium. *J. Mol. Biol.* **257,** 473–478 (1996).
4. Fukami-Kobayashi, K., Tateno, Y. & Nishikawa, K. Domain dislocation: a change of core structure in periplasmic binding proteins in their evolutionary history. *J. Mol. Biol.* **286,** 279–290 (1999).
5. Sakaguchi-Mikami, A., Taniguchi, A., Sode, K. & Yamazaki, T. Construction of a novel glucose-sensing molecule based on a substrate-binding protein for intracellular sensing. *Biotechnol. Bioeng.* **108,** 725–733 (2011).
6. Giuliani, S. E. *et al.* Environment sensing and response mediated by ABC transporters. *BMC Genomics* **12 Suppl 1,** S8 (2011).
7. Michalska, K. *et al.* Characterization of transport proteins for aromatic compounds derived from lignin: benzoate derivative binding proteins. *J. Mol. Biol.* **423,** 555–575 (2012).
8. Carmona, M. *et al.* Anaerobic catabolism of aromatic compounds: a genetic and genomic view. *Microbiol. Mol. Biol. Rev.* **73,** 71–133 (2009).
9. Díaz, E., Jiménez, J. I. & Nogales, J. Aerobic degradation of aromatic compounds. *Curr. Opin. Biotechnol.* **24,** 431–442 (2013).
10. Tsirigotaki, A., De Geyter, J., Šoštaric, N., Economou, A. & Karamanou, S. Protein export through the bacterial Sec pathway. *Nat. Rev. Microbiol.* **15,** 21–36 (2017).
11. Chen, X., Zaro, J. L. & Shen, W.-C. Fusion protein linkers: property, design and functionality. *Adv. Drug Deliv. Rev.* **65,** 1357–1369 (2013).
12. Thukral, S. K., Tavianini, M. A., Blumberg, H. & Young, E. T. Localization of a minimal binding

domain and activation regions in yeast regulatory protein ADR1. *Mol. Cell. Biol.* **9,** 2360–2369 (1989).

13. Durante-Rodríguez, G. *et al.* Identification of a missing link in the evolution of an enzyme into a transcriptional regulator. *PLoS One* **8,** e57518 (2013).
14. Wintjens, R. & Rooman, M. Structural classification of HTH DNA-binding domains and protein-DNA interaction modes. *J. Mol. Biol.* **262,** 294–313 (1996).
15. Aravind, L., Anantharaman, V., Balaji, S., Babu, M. M. & Iyer, L. M. The many faces of the helix-turn-helix domain: transcription regulation and beyond. *FEMS Microbiol. Rev.* **29,** 231–262 (2005).
16. Zhou, Y., Huang, H., Zhou, P. & Xie, J. Molecular mechanisms underlying the function diversity of transcriptional factor IclR family. *Cell. Signal.* **24,** 1270–1275 (2012).
17. Pennella, M. A. & Giedroc, D. P. Structural determinants of metal selectivity in prokaryotic metal-responsive transcriptional regulators. *Biometals* **18,** 413–428 (2005).
18. Gallegos, M. T., Schleif, R., Bairoch, A., Hofmann, K. & Ramos, J. L. Arac/XylS family of transcriptional regulators. *Microbiol. Mol. Biol. Rev.* **61,** 393–410 (1997).
19. Weickert, M. J. & Adhya, S. A family of bacterial regulators homologous to Gal and Lac repressors. *J. Biol. Chem.* **267,** 15869–15874 (1992).
20. Ramos, J. L. *et al.* The TetR family of transcriptional repressors. *Microbiol. Mol. Biol. Rev.* **69,** 326–356 (2005).
21. Ramot, R., Kishore Inampudi, K. & Wilson, C. J. Lactose repressor experimental folding landscape: fundamental functional unit and tetramer folding mechanisms. *Biochemistry* **51,** 7569–7579 (2012).
22. Chen, J. & Matthews, K. S. Deletion of lactose repressor carboxyl-terminal domain affects tetramer formation. *J. Biol. Chem.* **267,** 13843–13850 (1992).
23. de Kok, S. *et al.* Rapid and reliable DNA assembly via ligase cycling reaction. *ACS Synth. Biol.* **3,** 97–106 (2014).
24. Jacob, F. & Monod, J. Genetic regulatory mechanisms in the synthesis of proteins. *J. Mol. Biol.* **3,** 318–356 (1961).

25. Cherney, L. T., Cherney, M. M., Garen, C. R., Lu, G. J. & James, M. N. G. Crystal structure of the arginine repressor protein in complex with the DNA operator from Mycobacterium tuberculosis. *J. Mol. Biol.* **384,** 1330–1340 (2008).

26. Yokoyama, K. *et al.* Feast/famine regulatory proteins (FFRPs): Escherichia coli Lrp, AsnC and related archaeal transcription factors. *FEMS Microbiol. Rev.* **30,** 89–108 (2006).

27. Meinhardt, S. *et al.* Novel insights from hybrid LacI/GalR proteins: family-wide functional attributes and biologically significant variation in transcription repression. *Nucleic Acids Res.* **40,** 11139–11154 (2012).

28. Rigali, S., Derouaux, A., Giannotta, F. & Dusart, J. Subdivision of the helix-turn-helix GntR family of bacterial regulators in the FadR, HutC, MocR, and YtrA subfamilies. *J. Biol. Chem.* **277,** 12507–12515 (2002).

29. Krell, T., Molina-Henares, A. J. & Ramos, J. L. The IclR family of transcriptional activators and repressors can be defined by a single profile. *Protein Sci.* **15,** 1207–1213 (2006).

30. Muraoka, S. *et al.* Crystal structure of a full-length LysR-type transcriptional regulator, CbnR: unusual combination of two subunit forms and molecular bases for causing and changing DNA bend. *J. Mol. Biol.* **328,** 555–566 (2003).

31. Maddocks, S. E. & Oyston, P. C. F. Structure and function of the LysR-type transcriptional regulator (LTTR) family proteins. *Microbiology* **154,** 3609–3623 (2008).

32. Hobman, J. L., Wilkie, J. & Brown, N. L. A design for life: prokaryotic metal-binding MerR family regulators. *Biometals* **18,** 429–436 (2005).

33. Schreiter, E. R. & Drennan, C. L. Ribbon-helix-helix transcription factors: variations on a theme. *Nat. Rev. Microbiol.* **5,** 710–720 (2007).

34. Hall, D. R. *et al.* The high-resolution crystal structure of the molybdate-dependent transcriptional regulator (ModE) from Escherichia coli: a novel combination of domain folds. *EMBO J.* **18,** 1435–1446 (1999).

35. Madoori, P. K., Agustiandari, H., Driessen, A. J. M. & Thunnissen, A.-M. W. H. Structure of the transcriptional regulator LmrR and its mechanism of multidrug recognition. *EMBO J.* **28,** 156–166 (2009).

36. Durante-Rodríguez, G., Mancheño, J. M., Díaz, E. & Carmona, M. Refactoring the λ phage lytic/lysogenic decision with a synthetic regulator. *Microbiologyopen* **5,** 575–581 (2016).
