# Supplementary material for: Biosensor libraries harness large classes of binding domains for construction of allosteric transcriptional regulators
Source: Nat Commun. 2018 Aug 6;9:3101. doi: 10.1038/s41467-018-05525-6 (PMC6079105; doi:10.1038/s41467-018-05525-6)
Supplement: Supplementary file 3 — Description of Additional Supplementary Files [file 41467_2018_5525_MOESM3_ESM.pdf]

Description of Additional Supplementary Files

File Name: Supplementary Data 1
Description: A list of the main plasmids and bacterial strains used in this work

File Name: Supplementary Data 2
Description: All the oligonucleotides mentioned in the manuscript

File Name: Supplementary Data 3
Description: The collection of synthetic reporter promoters referred to in this paper
